# An Improved Extrapolation Scheme for Truncated CT Data Using 2D Fourier-Based Helgason-Ludwig Consistency Conditions

**DOI:** 10.1155/2017/1867025

**Published:** 2017-07-20

**Authors:** Yan Xia, Martin Berger, Sebastian Bauer, Shiyang Hu, Andre Aichert, Andreas Maier

**Affiliations:** ^1^Pattern Recognition Lab, Friedrich-Alexander-University Erlangen-Nuremberg, Erlangen, Germany; ^2^Erlangen Graduate School in Advanced Optical Technologies (SAOT), Erlangen, Germany; ^3^Siemens Healthcare GmbH, Forchheim, Germany

## Abstract

We improve data extrapolation for truncated computed tomography (CT) projections by using Helgason-Ludwig (HL) consistency conditions that mathematically describe the overlap of information between projections. First, we theoretically derive a 2D Fourier representation of the HL consistency conditions from their original formulation (projection moment theorem), for both parallel-beam and fan-beam imaging geometry. The derivation result indicates that there is a zero energy region forming a double-wedge shape in 2D Fourier domain. This observation is also referred to as the Fourier property of a sinogram in the previous literature. The major benefit of this representation is that the consistency conditions can be efficiently evaluated via 2D fast Fourier transform (FFT). Then, we suggest a method that extrapolates the truncated projections with data from a uniform ellipse of which the parameters are determined by optimizing these consistency conditions. The forward projection of the optimized ellipse can be used to complete the truncation data. The proposed algorithm is evaluated using simulated data and reprojections of clinical data. Results show that the root mean square error (RMSE) is reduced substantially, compared to a state-of-the-art extrapolation method.

## 1. Introduction

It is known that traditional computed tomography (CT) reconstruction algorithms, for example, filtered backprojection methods, are not compatible to laterally truncated projection data, which appears often in the case of either (1) when the object extends outside of the field of view (FOV) or (2) X-ray beam collimation for the purpose of dose reduction. If data truncation is not effectively compensated for, it will result in cupping-like artifacts and incorrect gray-value levels in the reconstruction. A typical approach to reduce truncation artifacts is to perform extrapolation, for example, with the symmetric mirroring method [1], water cylinder extrapolation method [2], optimization-based extrapolation scheme [3], or implicit extrapolation method performed in the second-order derivative domain [4]. However, these heuristic extrapolation methods typically rely on techniques that complete the truncated data by means of a continuity assumption and thus appear to be ad hoc.

It has been demonstrated that any physically consistent sinogram has a strong restriction in its functional form [5]. This restriction is expressed by Helgason-Ludwig (HL) consistency conditions [6, 7], which are a mathematical expression to precisely describe the overlap of information between different projections. The HL consistency conditions play an important role in image reconstruction from imperfect projection data (e.g., due to noise, motion, and truncation) since these projections no longer satisfy the HL conditions. Related work uses HL conditions to estimate motion parameters directly from sinograms [8–10] or to solve the problem of limited angle tomography using a variational formulation that incorporates HL conditions [11]. In PET/SPECT, the HL consistency conditions were also used for attenuation correction if no transmission data is available [12].

This work addresses consistency-based sinogram completion. The methods proposed in [2, 13] implicitly used the zeroth-order HL consistency condition; that is, the DC term is the same for all projections, as a constraint for data extrapolation. The first-order condition, which corresponds to the first moment of the projections and describes the so-called “center of mass,” was also used to guide the extrapolation procedure [14]. Later, the elliptical extrapolation suggested in [15] explicitly used a small subset of the consistency conditions in the original HL formulation (projection moment theorem) so that large numerical instability can be avoided when computing the moment terms. The approach in [16] modified this original formulation by expanding the Radon transform in terms of its basis functions and incorporated not only one or two HL consistency conditions, but theoretically an infinite number of such constraints. However, the HL consistency conditions proposed in [16] were represented in the Chebyshev-Fourier domain, which increased computational complexity for practical applications. To simplify the computation, the method in [17] refined the Chebyshev-Fourier representation of HL conditions using an FFT with additional cosine transform along the detector channel. Furthermore, fan-beam to parallel-beam rebinning is required since the consistency conditions were only derived for parallel-beam geometry.

In this paper, we first derive the HL consistency conditions in the 2D Fourier domain from their original formulation. The Fourier representation shows that there is a zero energy region appearing in the Fourier transform of a sinogram (symmetric for parallel-beam and asymmetric for fan-beam geometry). This property was also demonstrated in [18, 19], which is referred to as the Fourier property of a sinogram and which was approximately arrived at using the parallel-/fan-beam sinogram of a delta point object. If the projection data is imperfect or incomplete, the zero energy double-wedge region contains nonnegligible values that indicate the corresponding inconsistent components. Several applications using this Fourier property of a sinogram can be found in [8, 20–23]. In this work, we theoretically prove the equivalence between the HL consistency conditions and the Fourier property of a sinogram and show the advantages of applying these Fourier-based consistency conditions: first, an infinite number of conditions are considered; and second, 2D Fourier transform via FFT is computationally more efficient than the Chebyshev-Fourier transform [16] or Lagrange-Fourier transform [11]. These features allow us to develop an efficient data extrapolation method by optimization of a cost function based on the Fourier-based HL conditions. First investigation on the method was also reported in [24].

The organization of the paper is as follows. In Section 2, we review the HL consistency conditions in its original formulation and the modified Chebyshev-Fourier representation. Then, we derive 2D Fourier-based HL consistency conditions and extend the conditions from parallel-beam to fan-beam geometry for centered objects. In Section 3, we design a cost function based on HL consistency conditions, which we use in a constrained optimization over a uniform ellipse that describes the object outline. In Section 4, we present experimental results from both a simulated phantom and reprojections of clinical data. In Sections 5 and 6, we discuss the relevant issues and draw conclusion.

## 2. Consistency Conditions

### 2.1. Helgason-Ludwig (HL) Consistency Conditions

In this section, we review the original formulation of HL consistency conditions, which is also referred to as the projection moment theorem in the literature [16]. Suppose the object is supported on the unit disk centered at the origin. Let *a*_*n*_(*θ*) be the *n*th moment of the sinogram *g*(*θ*, *s*) with respect to the detector bin *s*, which is defined as

(1) $$\begin{array}{c} a_{n}\left(\;\theta \;\right)=\overset{1}{\underset{-1}{\int }}s^{n}g\left(\;\theta ,s\;\right)ds. \end{array}$$

Then, the function *a*_*n*_(*θ*) does not change arbitrarily when the rotation angle *θ* varies. The Fourier series expansion of *a*_*n*_(*θ*) can be written as follows:

(2) $$\begin{array}{c} a_{n}\left(\;\theta \;\right)=\overset{\infty }{\underset{k=0}{\sum }}a_{nk}\exp\left(\;jk\theta \;\right), \end{array}$$

with Fourier coefficients *a*_*nk*_ given by

(3) $$\begin{array}{c} a_{nk}=\frac{1}{2\pi }\overset{2\pi }{\underset{0}{\int }}a_{n}\left(\;\theta \;\right)\exp\left(\;-jk\theta \;\right)d\theta . \end{array}$$

Then, it is readily proven [25] that all *a*_*nk*_ necessarily satisfy

(4) $$\begin{array}{c} a_{nk}=0,\;\text{for }\left|k\right|>n. \end{array}$$

### 2.2. Chebyshev-Fourier Representation of HL Conditions

The derivation of the Chebyshev-Fourier version of HL conditions is similar to the one in [16]. But here we use the Chebyshev polynomial of the* first kind* to replace the monomial term *s*^*n*^, instead of the Chebyshev polynomial of the* second kind* as shown in [16].

Inserting (1) into (3) yields

(5) $$\begin{array}{c} a_{nk}=\frac{1}{2\pi }\overset{2\pi }{\underset{0}{\int }}\overset{1}{\underset{-1}{\int }}g\left(\;\theta ,s\;\right)s^{n}\exp\left(\;-jk\theta \;\right)ds\;d\theta . \end{array}$$

Note that the functions *s*^*n*^exp⁡(−*jkθ*) do not form a set of orthogonal basis functions on *L*_2_(*Z*, (1 − *s*^2^)^−1/2^) (the Radon transform maps the Hilbert space *L*_2_(*Ω*^2^) consisting of finite norm objects *f*(*x*, *y*) to the Hilbert space *L*_2_(*Z*, (1 − *s*^2^)^−1/2^) consisting of finite norm sinograms *p*(*θ*, *s*)), where −1 ≤ *s* < 1. In the following we show that the monomial *s*^*n*^ can be replaced by the *n*th-order orthogonal polynomial, such that the HL consistency conditions are tractable to use in reconstruction.

The sinogram *g*(*θ*, *s*) can be expanded in a series as follows:

(6) $$\begin{array}{c} g\left(\;\theta ,s\;\right)=\frac{1}{\pi }\overset{\infty }{\underset{n=-\infty }{\sum }}\;\overset{\infty }{\underset{k=0}{\sum }}b_{nk}{\left(\;1-s^{2}\;\right)}^{-1/2}T_{n}\left(\;s\;\right)\exp\left(\;jk\theta \;\right), \end{array}$$

where *b*_*nk*_ denote the expansion coefficients and *T*_*n*_(·) denotes the *n*th-order Chebyshev polynomial of the* first kind*, which is defined by

(7) $$\begin{array}{c} T_{n}\left(\;s\;\right)=\frac{n}{2}\overset{\left[n/2\right]}{\underset{i=0}{\sum }}c_{i}{\left(\;2s\;\right)}^{n-2i}=cos\left(\;n\arccos\left(\;s\;\right)\;\right), \end{array}$$

with

(8) $$\begin{array}{c} c_{i}=\frac{{\left(-1\right)}^{i}\left(n-i-1\right)!}{i!\left(n-2i\right)!}. \end{array}$$

Let *g*_1_, *g*_2_ ∈ *L*_2_(*Z*, (1 − *s*^2^)^−1/2^); we define the inner product of *g*_1_ and *g*_2_ as follows:

(9) $$\begin{array}{c} \langle \;g_{1},g_{2}\;\rangle =\overset{2\pi }{\underset{0}{\int }}\overset{1}{\underset{-1}{\int }}g_{1}\left(\;\theta ,s\;\right)g_{2}\left(\;\theta ,s\;\right)\sqrt{\left(1-s^{2}\right)}\;ds\;d\theta . \end{array}$$

From Appendix A we will prove that *T*_*n*_(*s*)(1 − *s*^2^)^−1/2^exp(*jkθ*) form an orthogonal basis of *L*_2_(*Z*, (1 − *s*^2^)^−1/2^).

Then, we can obtain an expression of the expansion coefficients *b*_*nk*_ as a scalar product

(10) bnkg,1πTns1−s2−1/2exp⁡−jkθ=1π∫02π∫−11gθ,sTnsexp⁡−jkθds dθ.

By comparing (5) and (10), it is noted that the coefficients *b*_*nk*_ are related to *a*_*nk*_ by the following combination:

(11) $$\begin{array}{c} b_{nk}=\frac{n}{2}\overset{\left[n/2\right]}{\underset{i=0}{\sum }}c_{i}2^{n-2i}a_{n-2i,k}. \end{array}$$

Then, we will have the HL conditions as

(12) k>n⟹k>n−2ii=0,1,…,n2⟹an−2i,k=0see  Eq. 4⟹bnk=0.

In sum,

(13) $$\begin{array}{c} b_{nk}=0,\;\text{if }\left|k\right|>n. \end{array}$$

### 2.3. 2D Fourier-Based HL Consistency Conditions

We perform the 2D Fourier transform to both sides of (6)

(14) Gη,ξ=1π∫02π∫−11∑n=−∞∞ ∑k=0∞bnk1−s2−1/2Tns×exp⁡jkθexp⁡−jηθ+ξsds dθ.

Because the term

(15) $$\begin{array}{c} \overset{\infty }{\underset{n=-\infty }{\sum }}\;\overset{\infty }{\underset{k=0}{\sum }}b_{nk}T_{n}\left(\;s\;\right)\exp\left(\;jk\theta \;\right)\exp\left(\;-j\left(\;\eta \theta +\xi s\;\right)\;\right) \end{array}$$

is uniformly convergent, the order of the integral operator and the sum operator can be changed:

(16) Gη,ξ=1π∑n=−∞∞ ∑k=0∞bnk∫02π∫−111−s2−1/2Tns×exp⁡jkθexp⁡−jηθ+ξsds dθ=1π∑n=−∞∞ ∑k=0∞bnk∫−111−s2−1/2Tnsexp⁡−jξsds×∫02πexp⁡−jη−kθdθ.

Because of the orthogonality of complex exponentials, we know that

(17) $$\begin{array}{c} \overset{2\pi }{\underset{0}{\int }}\exp\left(\;-j\left(\;\eta -k\;\right)\theta \;\right)d\theta =\left\{\begin{array}{cc} 0, & \eta \neq k,\\ 2\pi , & \eta =k. \end{array}\right. \end{array}$$

Then, we have

(18) Gη,ξ=2∑n=−∞∞bnη∫−111−s2−1/2Tnsexp⁡−jξsds=2∑n=−∞∞bnηJn−ξ2−jnsee  Appendix  B,

where *J*_*n*_ is the* first kind* Bessel function of order *n*.

According to Debye's relation [25], we know that *J*_*n*_(*ξ*) decays exponentially when |*n*| > |*ξ*|. From (13) we also know that *b*_*nη*_ = 0 when |*η*| > *n*. Thus, we will have 2D Fourier representation of the HL condition as follows:

(19) $$\begin{array}{c} G\left(\;\eta ,\xi \;\right)\approx 0,\;\text{for }\left|\eta \right|>\xi . \end{array}$$

So far, we only assume that the object is supported on the unit disk. If the object is supported by a disk with a radius *r*, then we replace *s* by *s*′ = *s*/*r* (where |*s*′| ≤ 1) in (14) and (18) such that we can obtain the following conditions:

(20) $$\begin{array}{c} G\left(\;\eta ,\xi \;\right)\approx 0,\;\text{for }\left|\eta \right|>r\xi , \end{array}$$

where *r* is the largest object support. Note that this property was also found in [18] by investigating the parallel-beam sinogram of a point object.

**Figure 1:**
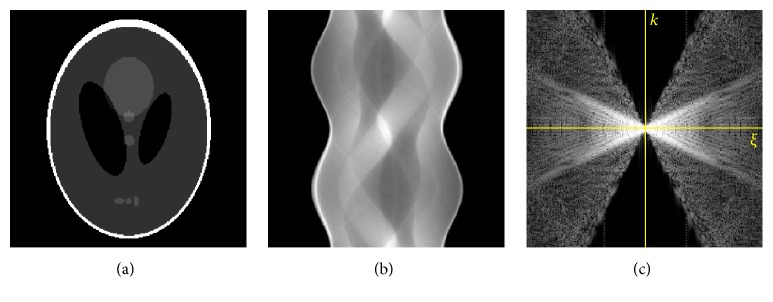
Illustration of zero coefficients in the 2D Fourier transform of a sinogram. (a) Shepp-Logan phantom, (b) the corresponding sinogram, and (c) the 2D Fourier transform of the sinogram.

Figure 1 illustrates a double-wedge region of zero coefficients in the 2D Fourier transform of a sinogram, when |*η*| > *rξ*.

### 2.4. HL Conditions in Fan-Beam Geometry

With variable substitution, that is, *θ* = *β* + *α*, *s* = *R*sin⁡*α* (*β* is the rotation angle in fan-beam geometry, *α* is the opening fan angle, and *R* is the source-to-isocenter distance), the projection moment theorem (i.e., (5)) in fan-beam notation can be expressed as

(21) ank12π∫02π∫−π/2π/2gFanβ,αRsin⁡αn·exp⁡ikβ+αdβ dRsin⁡α,

where

(22) $$\begin{array}{c} a_{nk}=0,\;\text{for }\left|k\right|>n. \end{array}$$

Similar to the parallel-beam case, we consider the object support is *r* and expand *g*_Fan_(*β*, *α*) in the Chebyshev-Fourier space as

(23) gFanβ,α=1π∑n=−∞∞ ∑k=0∞bnkTnRsin⁡α/r1−Rsin⁡α/r2exp⁡jkβ+α.

Also, we readily obtain the relation *b*_*nk*_ = 0, for |*k*| > *n*. Then, 2D Fourier transform of both sides of (23) and simplification according to (17) yield

(24) GFanη,m=1π∫02π∫−π/2π/2∑n=−∞∞ ∑k=0∞bnηTnRsin⁡α/r1−Rsin⁡α/r2×exp⁡jkβ+αexp⁡−jηβ+mαdα dβ=1π∑n=−∞∞bnη∫−π/2π/2TnRsin⁡α/r1−Rsin⁡α/r2×exp⁡jη−mαdα.

According to (B.2) in Appendix B, the innermost integral with respect to *α* becomes

(25) ∫−π/2π/2TnRsin⁡α/r1−Rsin⁡α/r2exp⁡jη−mαdα=12−jn·∫−π/2π/2∫−∞∞exp⁡−jξRsin⁡αJnrξdrξ·exp⁡jη−mαdα=r2−jn∫−∞∞Jnrξ·∫−π/2π/2exp⁡−jξRsin⁡α+jη−mαdα dξ=r2−jn∫−∞∞JnrξJη−mRξdξ.

It is known that the Weber-Schafheitlin's integral *J*_*n*_(*rξ*)*J*_*η*−*m*_(*Rξ*) decays very fast for *R*|*n*| > *r*|*η* − *m*| [25]. Together with the relation *b*_*nη*_ = 0, for |*η*| > *n*, we finally arrive at

(26) $$\begin{array}{c} G_{Fan}\left(\;\eta ,m\;\right)\approx 0,\;\text{for }R\left|\eta \right|>r\left|\eta -m\right|. \end{array}$$

For an equally spaced fan-beam geometry, we apply the 2D Fourier transform to (23) with respect to *β* and *u*:

(27) GFanη,l=1π∫02π∫−∞∞∑n=−∞∞ ∑k=0∞bnkTnRsin⁡α/r1−Rsin⁡α/r2·eikβ+αe−jηβ+ludu dβ,

where *u* = *D*tan⁡*α* and *D* denotes the source-detector distance.

Since *D* is large compared to *u* (for a small fan angle), we can make an approximation *u* = *Dα*. Then, we will have a similar derivation as for (25):

(28) ∫−π/2π/2TnRsin⁡α/r1−Rsin⁡α/r2exp⁡jη−lDαdDα=rD2−jn∫−∞∞JnrξJη−lDRξdξ.

Finally, we will obtain the HL conditions for an equally spaced fan-beam geometry:

(29) $$\begin{array}{c} G_{Fan}\left(\;\eta ,l\;\right)\approx 0,\;\text{for }R\left|\eta \right|>r\left|\eta -lD\right|. \end{array}$$

Note that both (26) and (29) were approximately arrived at in [19] when investigating the fan-beam sinogram of a delta function point.

## 3. Data Extrapolation Using HL Consistency Conditions

**Figure 2:**
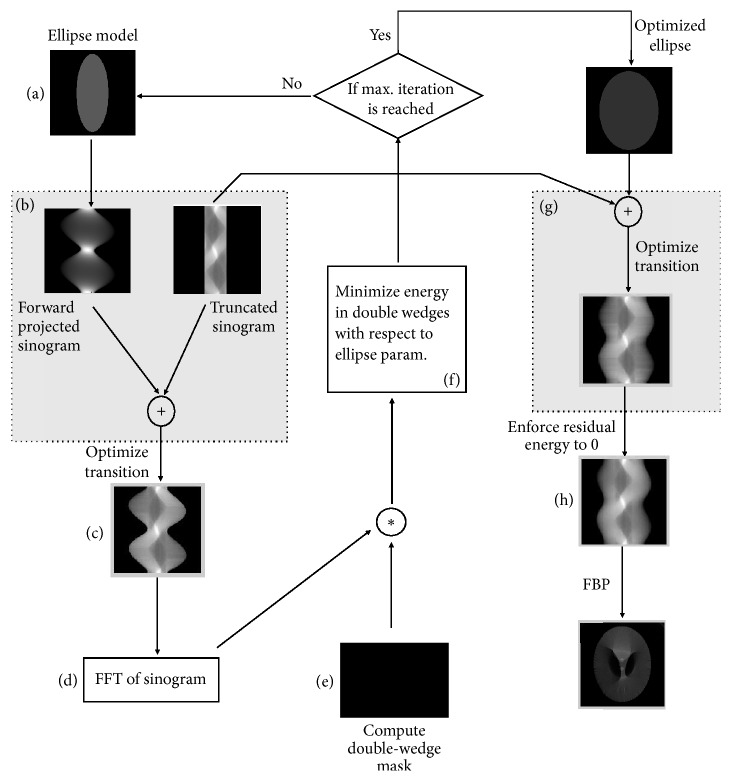
Flowchart of the proposed sinogram completion scheme: Set up an ellipse model for sinogram completion and optimize the model parameters (here the two ellipse radii) by enforcing the Fourier constraints.

The HL consistency conditions play an important role in image reconstruction as they can be used as a measure to restore consistency of the imperfect projection data (e.g., due to noise, motion, or truncation). In this work, we take advantage of the theory derived before for data extrapolation with truncated projection data. A flowchart of our proposed projection completion algorithm can be described as follows (also see Figure 2 for illustration).

**Step 1 .**

Segment the double-wedge region in the 2D Fourier transform of the sinogram as shown in Figure 1(c). Then, compute the slope of double wedge, that is, *η*/*ξ* at double-wedge's edge. The object support *r* can be computed using (20); that is, *r* = |*η*|/*ξ*, for instance, for a parallel-beam sinogram.

**Step 2 .**

Set up a shape model for sinogram completion (detruncation) and fit the model to the measured (truncated) data with a detruncation optimization algorithm that enforces the constraint that the values within the double-wedge region of the Fourier transformed sinogram are zero.

(a) Set up a uniform ellipse model *f*(*μ*, *a*, *b*, *x*_0_, *y*_0_, *ϕ*) and initialize the model with two radii (*a*, *b*). For reasons of simplicity, assume a uniform density of the measured object. The density *μ* can be determined by two ways: (1) heuristic preset, for example, water density; (2) extrapolating the sinogram up to the object support, reconstructing it using an FBP framework, and using the mean of the reconstructed object as the density value. Additionally, current consistency conditions are only derived for a centered object; we assume the ellipse center (*x*_0_, *y*_0_) = (0,0).
(b) Complete the truncated/measured sinogram *g*(*θ*, *s*) using forward projections of the ellipse model. Here the forward projections only need to be computed for the regions outside of the scan's field of view.
  (30) $$\begin{array}{c} g_{combined}\left(\;\theta ,s\;\right)=g\left(\;\theta ,s\;\right)+Rf\left(\;\mu ,a,b,x_{0},y_{0},\phi ,\theta ,s\;\right), \end{array}$$
where *R* indicates the Radon Transform.
(c) Optimize (adapt/smooth) the transition region of the completed sinogram by adding/subtracting an offset *d*_*n*_(*θ*) to each row of the forward-projected sinogram. The offset values *d*_*n*_(*θ*) are computed by comparing two neighboring pixels from the forward-projected and the measured sinogram as follows:
  (31) $$\begin{array}{c} d_{n}\left(\;\theta \;\right)=\left|\;g\left(\;\theta ,n\;\right)-Rf\left(\;\mu ,a,b,x_{0},y_{0},\phi ,\theta ,n\;\right)\;\right|, \end{array}$$
where *n* indicates the index of neighboring pixels between the extrapolated and the measured sinogram. Note that this rescaling/intensity adjustment of the forward-projected sinogram also weakens the impact of the ellipse density such that it is not mandatory to set the density as an additional optimization parameter.
(d) Perform 2D fast Fourier transform (FFT) of the combined sinogram *G*(*η*, *ξ*) = *F*[*g*_combined_(*θ*, *s*)].
(e) Sum up the energies in the double-wedge region *E*(*μ*, *a*, *b*, *x*_0_, *y*_0_, *ϕ*)
  (32) $$\begin{array}{c} E\left(\;\mu ,a,b,x_{0},y_{0},\phi \;\right)=\underset{\left(\eta ,\xi \right)\in \Omega_{DW}}{\sum }\left|\;G\left(\;\eta ,\xi \;\right)\;\right|, \end{array}$$
where *Ω*_DW_ denotes the double-wedge region.
(f) Minimize the energy in the double-wedge region by optimizing the ellipse parameters **p** = (*μ*, *a*, *b*, *x*_0_, *y*_0_, *ϕ*)
  (33) $$\begin{array}{c} p=arg\;\underset{p}{min}\left(\;E\left(\;p\;\right)\;\right). \end{array}$$
(g) Based on the estimated ellipse parameters **p**, perform the sinogram completion and apply transition smoothing, as already described in (c).
(h) Perform 2D FFT, set the residual energy in the double-wedge region to zero (hard constraint), and perform an inverse 2D FFT. To improve the reconstruction result, this step can be performed in an iterative manner.
  (34) $$\begin{array}{c} g_{optimized}\left(\;\theta ,s\;\right)=F^{-1}\left\{\;G\left(\;\eta ,\xi \;\right)=0\;|\;\left(\;\eta ,\xi \;\right)\in \Omega_{DW}\;\right\}. \end{array}$$

**Step 3 .**

The resulting completed (detruncated) sinogram (being optimized in the Fourier domain) is reconstructed using any reconstruction algorithm that can be applied on nontruncated projection data, for example, the standard FBP framework.

The detection to the wedge region in Step 1 can be performed using edge-based segmentation with known properties such as line-symmetric for parallel-beam sinogram and point-symmetric for fan-beam case. For proof of concept, in current work we assume that object support *r* is known and thus the wedge region can be directly computed using (29). Then, in (e) we set up a binary double-wedge mask with zero entries outside the double-wedge region and one within the double-wedge region. After applying the mask (element-wise multiplication) on the Fourier transformed completed sinogram from (d), we can sum up the energies in the double-wedge region.

For the task of minimization in (f), we use a Differential Evolution (DE) optimization [26] to search large spaces of candidate solutions and avoid the local minima. It is a stochastic, population-based global optimization method that appears fairly fast and robust for nondifferentiable and nonlinear objective functions. It uses a fixed number *N* of parameter vectors as a population for each iteration (also referred to as a generation). Firstly, the trial parameter vectors (parent) are initialized on an interval which defines upper and lower bounds of parameters. At each iteration/generation, every parent is combined one by one with a set of new vectors which results in a set of trial vectors (children). These newly generated children vectors are mixed with a predetermined target vector with probability CR, generating the new trial vector. Finally, the new trial vector replaces the “least useful” parent that yields the largest cost function value if and only if its cost function value is lower than that of this parent. The steps above continue until some stopping criterion is reached. In this work, we choose Scheme DE 1 proposed in [26] with parametrization as *N* = 20, *F* = 0.8, and CR = 0.7. We finish the optimization procedure if a preset maximum iteration number is reached.

## 4. Experiments and Results

The proposed method was validated and evaluated on the Shepp-Logan phantom and reprojected clinical data (data courtesy of CHI St. Luke's Health-Baylor St. Luke's Medical Center, Houston, TX, USA). All datasets were virtually collimated (by setting the area outside of the region of interest to zero) to a medium FOV and a small FOV. An equally spaced fan-beam imaging geometry is used. The sinogram of the Shepp-Logan phantom consists of 720 channels and 360 views over 360° rotation angle (full scan). Then, the phantom was reconstructed in a 256 × 256 matrix with the radius of the medium ROI being 60 pixels and the radius of the small ROI being 30 pixels. The clinical data was forward-projected to a sinogram consisting of 1500 channels and 720 views over 360° rotation angle (full scan). The forward-projected sinogram was reconstructed in a 512 × 512 matrix. The radius of the medium ROI is 95 pixels and the radius of the small ROI is 60 pixels. We also investigate the performance of the state-of-the-art water cylinder extrapolation method [2] and compare it with our proposed method.

**Figure 3:**
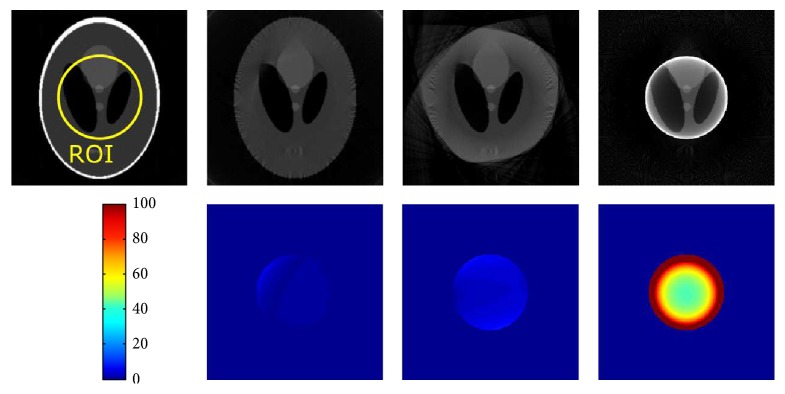
Reconstruction results of Shepp-Logan phantom (radius of ROI: 60 pixels). From left to right: reference from nontruncated data, proposed correction method, water cylinder extrapolation (cf. Hsieh et al. [2]), and no correction. The grayscale window is [0,1].

**Figure 4:**
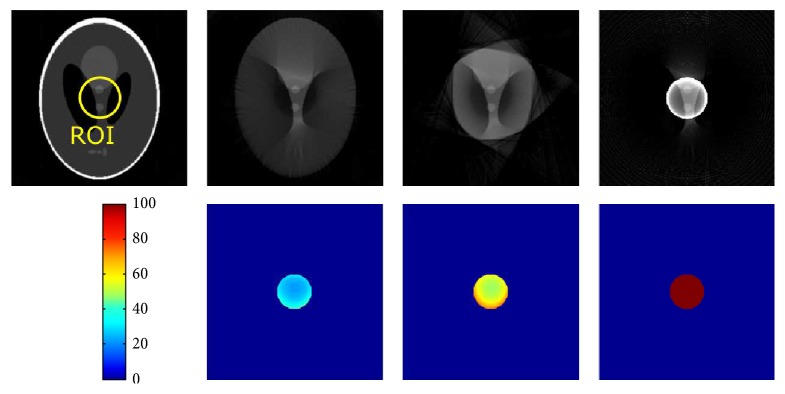
Reconstruction results of Shepp-Logan phantom (radius of ROI: 30 pixels). From left to right: reference from nontruncated data, proposed correction method, water cylinder extrapolation (cf. Berger et al. [8]), and no correction. The grayscale window is [0,1].

**Figure 5:**
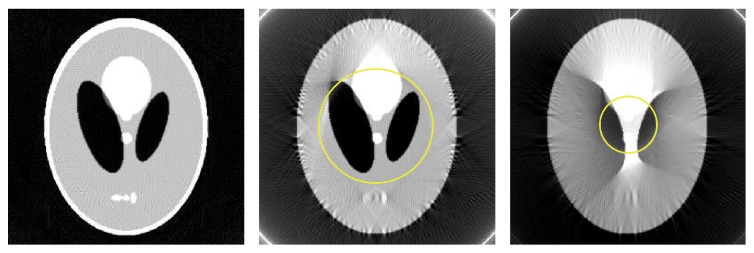
Reconstruction results of Shepp-Logan phantom using the proposed extrapolation method. From left to right: reference from nontruncated data, proposed correction method with medium truncation, and proposed correction method with severe truncation. The grayscale window is [0.1,0.2]. The yellow circles indicate the ROI.

Shown in Figures 3 and 4 are the reconstruction results from the Shepp-Logan phantom with different degrees of truncation (medium/small FOV). Difference images with respect to a reference FBP reconstruction from the original nontruncated data are also presented. The grayscale values are normalized into an arrangement of [0,1]. As expected, reconstruction without any correction will generate typical cupping-like truncation artifacts and a substantial offset compared to the reference. These artifacts, on the other hand, are effectively compensated by using water cylinder extrapolation as well as the proposed method, yielding only a small error spreading over the difference images. Note that, for the Shepp-Logan phantom with a rather simple structure, the proposed method is able to accurately estimate the outline of the object, in contrast to inferior shape estimation from the water cylinder extrapolation scheme, in which extrapolation is performed by fulfilling the continuity assumptions.  Figure 4 shows the case of severe truncation, where in general the reconstruction bias becomes larger as less data can be used for robust extrapolation and thus is more challenging for truncation correction algorithms. In this case, the reconstruction from the proposed method obviously shows less bias and truncation artifacts than that of the water cylinder extrapolation, while still retaining an accurate shape estimation. We also show the reconstruction images in a compressed window [0.1,0.25] (see Figure 5) to demonstrate the robustness of the proposed method to both medium truncation and severe truncation case.

**Figure 6:**
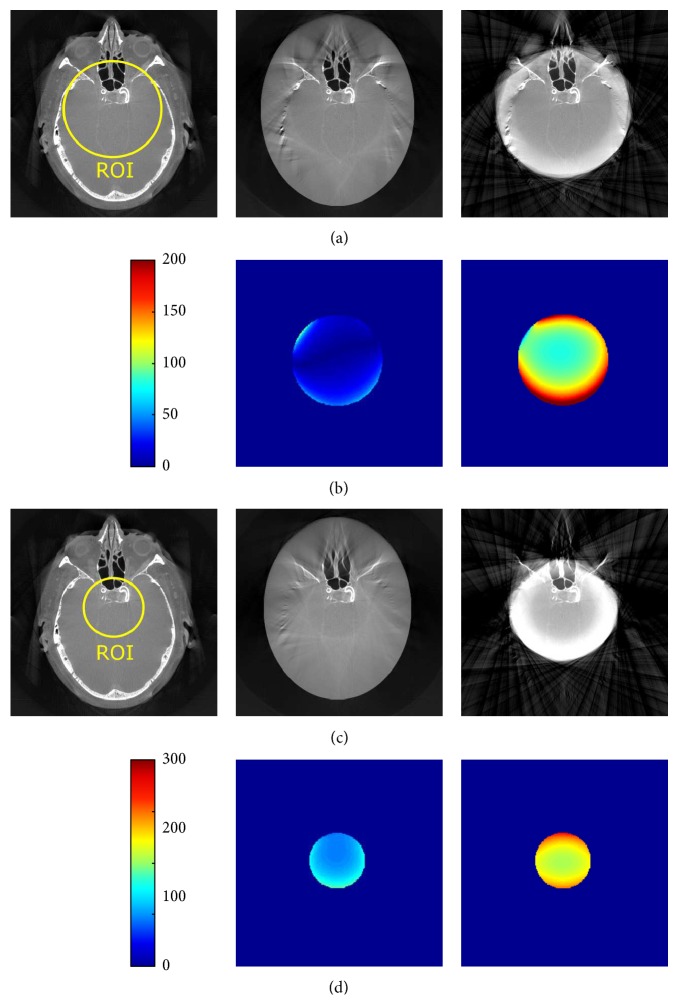
Reconstruction results of the reprojected clinical dataset 1 with both medium truncation (a) and severe truncation (c). (b) and (d) show the corresponding difference images with respect to the reference (only computed in the ROI region). From left to right: reference from nontruncated data, proposed correction method, and water cylinder extrapolation (cf. Berger et al. [8]). The grayscale window: *C* = 0 HU, *W* = 2000 HU.

**Figure 7:**
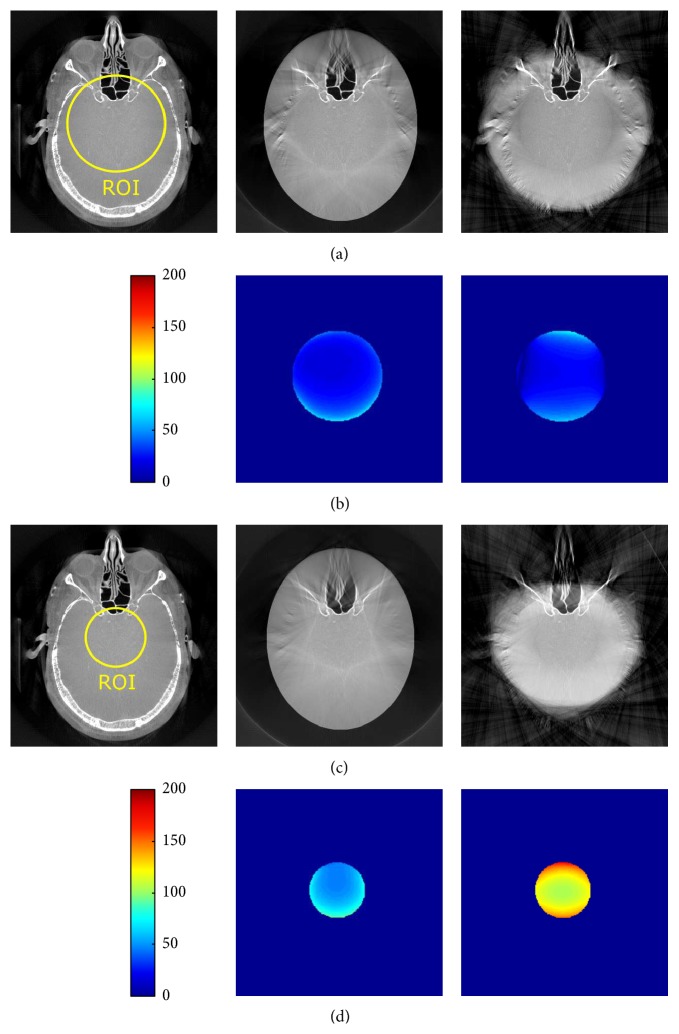
Reconstruction results of the reprojected clinical dataset 2 with both medium truncation (a) and severe truncation (c). (b) and (d) show the corresponding difference images with respect to the reference (only computed in the ROI region). From left to right: reference from nontruncated data, proposed correction method, and water cylinder extrapolation (cf. Berger et al. [8]). The grayscale window: *C* = 0 HU, *W* = 2000 HU.

**Figure 8:**
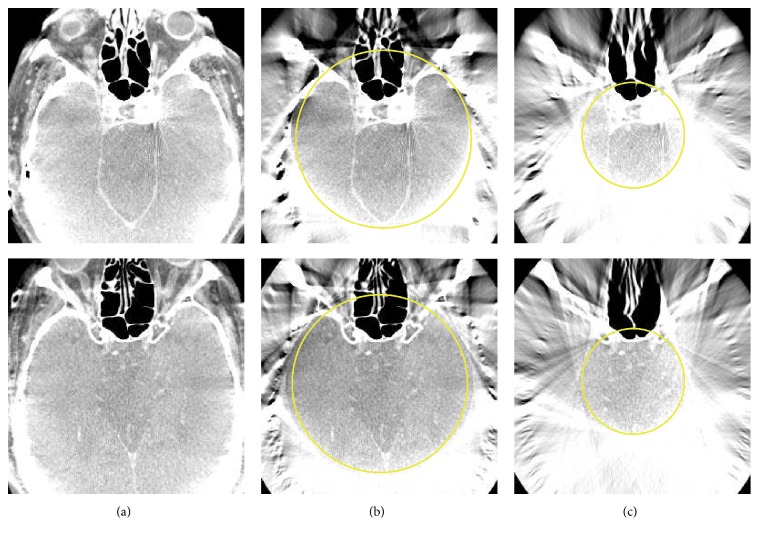
Reconstruction results of the reprojected clinical datasets 1 and 2 with both medium truncation (b) and severe truncation (c). Yellow circle indicates the ROI. The grayscale window: *C* = 0 HU, *W* = 400 HU.

**Figure 9:**
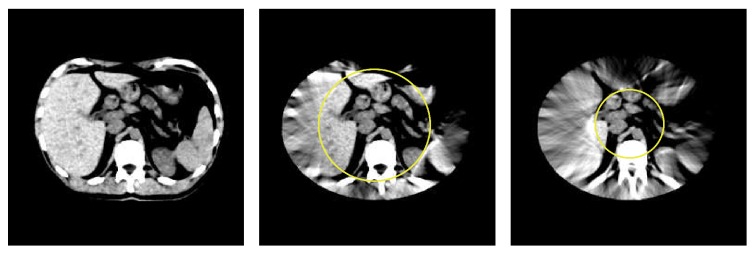
Reconstruction results of the reprojected body scan with both medium truncation and severe truncation. From left to right: the reference from nontruncated data, the proposed correction method with medium truncation, and the proposed correction method with severe truncation. The grayscale window: *C* = 0 HU, *W* = 400 HU. The yellow circles indicate the ROI.

**Figure 10:**
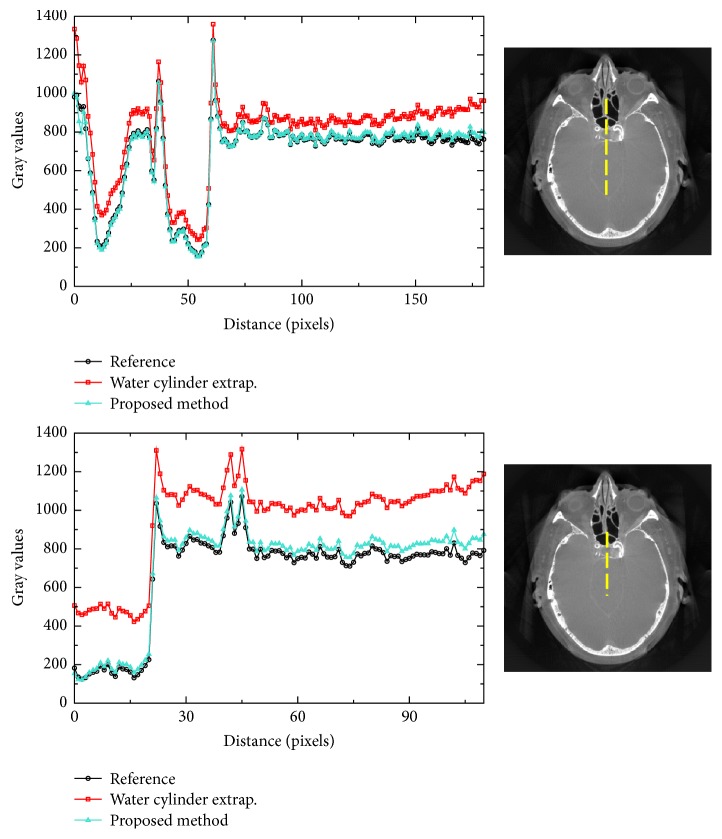
Plots of line profiles for each algorithm in the medium truncation case and in the severe truncation case for clinical dataset 1. The yellow dashed line in the corresponding right image indicates the position of the line profile.

**Figure 11:**
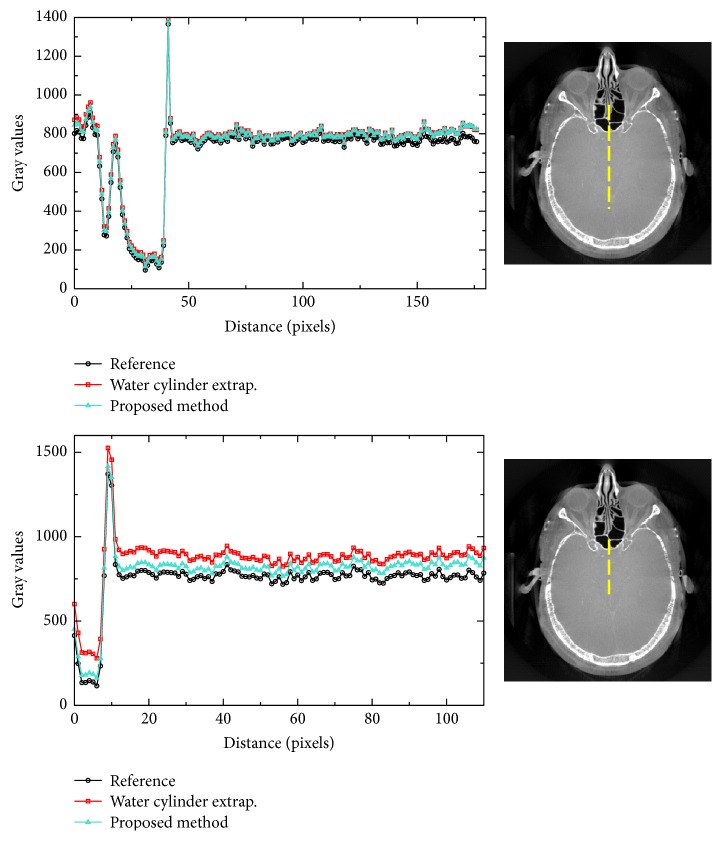
Plots of line profiles for each algorithm in the medium truncation case and in the severe truncation case for dataset 2. The yellow dashed line in the corresponding right image indicates the position of the line profile.

The results for reprojected clinical data are shown in Figures 6 and 7, also using two different degrees of truncation. The same reconstructions but with a compressed display window are shown in Figure 8. We can see that, for medium truncation (Figures 6(a) and 7(a)), both water cylinder extrapolation and the proposed method are able to reconstruct the image with high quality within the ROI: no typical cupping artifact and obvious bias are observed. However, when it comes to severe truncation, the water cylinder extrapolation performance degrades more than the performance of the proposed method.  Figure 9 shows reconstructions of a reprojection from a slice of body scan, which basically have a similar observation to that of head scan. The line profiles in Figures 10 and 11 show that a bias always appears in water cylinder extrapolation results compared to the reference but is less observed in the proposed method. Quantitative results in Table 1 confirm that the performance of the water cylinder extrapolation appears to be ad hoc. It yields a root mean square error (RMSE) gray value of 271.6 while for the proposed method the error is always lower than 60 gray values. The proposed method is also able to nicely recover the structural information of the object and reduces the cupping artifacts. Thus, it yields a high correlation coefficient (CC) with respect to the reference, that is, 0.99, compared to a CC of 0.98 for the water cylinder extrapolation scheme.

## 5. Discussion

The Fourier-based Helgason-Ludwig consistency conditions are derived for both parallel-beam and fan-beam geometry, as described in (20), (26), and (29). The derivation outcomes indicate the Fourier property of a physically consistent sinogram: there are zero coefficients forming a double-wedge region (symmetric for parallel-beam and point-symmetric for fan-beam geometry) in its 2D Fourier transform. Interestingly, the same property was also observed previously in the literature by investigating the parallel-/fan-beam sinogram of a delta point object [18, 19]. In [19], the authors clarify that the approximation to a Bessel function (see Eq. (8) in [19]) was arrived at intuitively and is validated empirically.

Motivated by these previous practical observations, we generate theoretical derivation. Our derivation stems directly from the original formulation of HL consistency conditions and is theoretically exact for parallel-beam geometry as well as equal-angle fan-beam geometry. In case of an equally spaced fan-beam case, such a derivation is not straightforward. To derive a similar property, we made an approximation that *u* = *Dα* under the assumption that the opening fan angle is small. Therefore, there could potentially be a misestimation of zero-energy region for large fan angles, as also observed in [19].

The benefits of Fourier-based consistency conditions are as follows: First, rather than using a small subset of the consistency conditions as proposed in [2, 13–15], an infinite number of conditions are implicitly considered in the Fourier representation. Second, the 2D Fourier transform via FFT is computationally more efficient than other transforms (e.g., the Chebyshev-Fourier transform [16] or Lagrange-Fourier transform [11]). This allows us to develop more efficient sinogram recovery schemes, as demonstrated in this paper.

The sinogram-based extrapolation scheme we proposed in this work incorporates the Fourier-based consistency conditions as a constraint for optimizing the ellipse parameters so that the missing data can be more accurately fitted. Experiments on both phantom and clinical data yielded promising results. There are some limitations to this Fourier constrained extrapolation method applied to ROI reconstruction. First, the current derivation only involves the sinogram of a centered object. It is not clear how the zero energy region will change for off-center cases. Second, for evaluation we used a full scan fan-beam geometry. We observed that, for a short scan acquisition where projection data is acquired only over a range of *π* plus the fan angle, some nonzero values also appear in zero energy region, which may affect the optimization procedure. Thus, corresponding consistency conditions that also account for an off-centered object and short scan acquisition would be interesting for future work. Also, in this work we used a uniform ellipse model to generate the sinogram outside measured region. Such an assumption is well suited for the imaged objects that can be approximated by a single ellipse, for example, head scan. For complicated objects such as knee scan, multiple ellipses may be superposed and optimized.

## 6. Conclusion

In this paper, we theoretically derived the 2D Fourier-based Helgason-Ludwig consistency conditions that can be evaluated very efficiently via FFT. Then, we suggested a sinogram-based extrapolation scheme that incorporates these consistency conditions as a constraint for optimizing the ellipse parameters. Then, the forward projection of the optimized ellipse can be used to complete the truncation data. Experiments on both phantom and clinical data yielded promising results of the proposed approach. The reconstruction results indicate that the proposed approach substantially outperforms a conventional water cylinder extrapolation approach [2], particularly for severe truncation, regarding both image quality and residual artifacts.

## Figures and Tables

**Table 1:** Summary of quantitative evaluation of truncation correction methods for medium truncation (ROI: radius of 95 pixels) and severe truncation (ROI: radius of 60 pixels).

	Algorithms	Head scan 1		Head scan 2	
		Medium	Severe	Medium	Severe
RMSE	Water cylinder extrapolation	123.0	271.6	29.7	114.2
	Proposed method	20.1	43.4	29.3	53.3
CC	Water cylinder extrapolation	0.987	0.985	0.996	0.984
	Proposed method	0.996	0.995	0.997	0.990

## Appendix

### A. Proving That *T*_*n*_(*s*)(1 − *s*^2^)^−1/2^exp⁡(*jkθ*) Form an Orthogonal Basis of *L*_2_(*Z*, (1 − *s*^2^)^−1/2^)

(A.1) Tns1−s2−1/2exp⁡jkθ,Tn∗s1−s2−1/2·exp⁡jk∗θ=∫02π∫−11Tns1−s2−1/2exp⁡jkθ×Tn∗s·1−s2−1/2exp⁡jk∗θ·1−s21/2ds dθ=∫−11TnsTn∗s1−s2−1/2·∫02πexp⁡jkθexp⁡jk∗θ=δnn∗δkk∗.

### B. Relationship between Bessel Function and Chebyshev Polynomial

The Fourier transform of the Bessel function can be computed as

(B.1) $$\begin{array}{c} \overset{\infty }{\underset{-\infty }{\int }}\exp\left(\;-j\xi s\;\right)J_{n}\left(\;s\;\right)ds=\frac{2{\left(-j\right)}^{n}}{{\left(1-\xi^{2}\right)}^{1/2}}T_{n}\left(\;\xi \;\right), \end{array}$$

for *ξ*^2^ < 1. We can swap the variables *s* and *ξ* and move the term 2(−*j*)^*n*^ to the left side:

(B.2) ∫−∞∞exp⁡−jξsJnξ12−jndξ=1−s2−1/2Tns,

where *s*^2^ < 1.

Then, substituting *ξ* by −*ξ* and computing the Fourier transform of both sides give

(B.3) $$\begin{array}{c} \frac{J_{n}\left(-\xi \right)}{2{\left(-j\right)}^{n}}=\overset{\infty }{\underset{-\infty }{\int }}{\left(\;1-s^{2}\;\right)}^{-1/2}T_{n}\left(\;s\;\right)\exp\left(\;-j\xi s\;\right)ds. \end{array}$$
